# Exploring the determinants of exclusive breastfeeding among infants under six months in the Gambia using gambian demographic and health survey data of 2019-20

**DOI:** 10.1186/s12884-023-05544-2

**Published:** 2023-04-01

**Authors:** Bewuketu Terefe, Kegnie Shitu

**Affiliations:** 1 Department of Community Health Nursing, School of Nursing, College of Medicine and Health Sciences, University of Gondar, Po. Box: 196 Gondar, Ethiopia; 2 Department of Health Education and Behavioral Science, Institute of Public Health, College of Medicine and Health Sciences, University of Gondar, Gondar, Ethiopia

**Keywords:** Exclusive breastfeeding, Infants under six months, Determinants, Gambia

## Abstract

**Introduction:**

For infants, no one is as nutritious as breastmilk for the rest of their lives. It is a great guarantee for their future health, especially if they can exclusively breastfeed for the next few months, from the moment they are born until the end of the fifth month. Although breastfeeding rates are very low, there is no data record about it in the Gambia.

**Objective:**

This study aimed to assess the status of exclusive breastfeeding and its determinants among infants under six months of age in the Gambia.

**Methods:**

It is a secondary data analysis using the 2019–20 Gambia demographic and health survey data. A total of 897 weighted mother-infant paired samples were included in the study. A logistic regression analysis method was employed to declare factors significantly associated with exclusive breastfeeding among infants under six months of age in Gambia. Variables with a p-value of 0.2 were entered into multiple logistic regression analysis, and after controlling other confounding factors, an adjusted odds ratio of 95% CI was applied to identify associated variables.

**Results:**

Exclusive breastfeeding was found in 53.63% only among infants under six months of age. Being a rural resident (AOR = 2.14, 95% CI: 1.33, 3.41), reading a newspaper (AOR = 5.62, 95% CI: 1.32, 24.09), and being counseled on breastfeeding by a health professional (AOR = 1.36, 95% CI: 1.01, 1.82) are times more likely to practice exclusive breastfeeding, respectively. On the other hand, a child with a fever (AOR = 0.56, 95% CI: 0.37, 0.84), a child whose age is 2–3 months (AOR = 0.41, 95 CI: 0.28, 0.59), and a child whose age is 4–5 months (AOR = 0.11, 95% CI: 0.07, 0.16) is less likely to be fed exclusively than a 0–1-month-old child.

**Conclusion:**

Exclusive breastfeeding remains among the public health challenges in the Gambia. Strengthening health professionals’ counseling techniques on breastfeeding and infant illnesses, promoting the pros of breastfeeding, and designing timely policies and interventions are urgently needed in the country.

## Introduction

Exclusive breastfeeding (EBF) is defined by the World Health Organization (WHO) as the state of affairs where the infant has fed only breast milk during their first six months of life from their mother or a wet nurse or expressed breast milk and no other additional liquids or solids, except for drops or syrups consisting of vitamins, mineral supplements, or medicines [1]. WHO’s recent report stated that, although exclusive breastfeeding contributes significantly to the overall development and growth of infants, over 66% of the world’s infants have not been breastfed exclusively for the past two decades, especially in less developed nations where the problem is more challenging [2, 3]. In the region of America, only 38% of babies are exclusively breastfed until the age of six months [4]. EBF has a massive impact on morbidity, mortality, and a mother’s health [5]. The world will have a chance of protecting more than 1.4 million under-five deaths through best EBF practices every single year [6]. Globally, of the total 8.2 million under-five deaths, more than 40% of them are caused by infections, and around 99% of deaths occur in developing nations, especially in Africa and Asia [7]. Beyond preventing death, EBF provides a strong mother-to-child relationship, reducing various childhood illnesses, including infections, diarrhea, pneumonia, diabetes mellitus, malocclusion, and the like [5, 8, 9]. In addition to its prevention of cancer, it also provides better education performance, productivity, and intellectual development to children [5, 8–10]. The importance of EBF is not limited to children but also prevents nursing mothers from experiencing depression, various cancers, hemorrhage, and it keeps them by regulating their weight [8, 11]. Therefore, enhancing mothers’ knowledge and attitude will have a great positive impact on the process of EBF practices [12, 13].

Despite many researchers advocating that EBF is crucial to produce a healthy future generation,

[14, 15], Western African countries are among the top list in their low rate of EBF practice [16–18]. Due to this millions of children have been dying before they reach five years old, however, it could be possible to save more than 200,000 lives annually in western Africa alone by practicing good EBF [16, 19]. A mapping study that included all African nations from 2000 to 2017 showed that the overall prevalence rate of EBF was 37%, which needs a massive acceleration of improvement to achieve the WHO recommendation [20]. This chronic public burden is likely to be a major impediment to the world health assembly’s (WHA) plan of reaching breastfeeding coverage by 50% in 2012–2025 [21]. Substantial studies have been conducted in western African countries at various time intervals to identify factors that could influence EBF practice. Educational status, type of residency, wealth index, birthing, birth order, vising of ANC and PNC, types of birthing, delivery assistance, age, and occupational status were among the topic individual and community level factors associated with EBF practice [22–24].

In the Gambian context, only a few are known related to EBF practice. An institutional-based cross-sectional study conducted in the Gambia showed that women’s knowledge, attitude, and counseling by health care providers on EBF were 69.2%, 38.6%, and 34.4% [25]. Another study revealed that 57% of neonatal died by infection and 84% of them died without receiving any treatment [26]. According to the report of the Gambian demographic and health survey of 2019/20, infant and neonatal mortality have increased from 34 to 42/1000 and 22 to 29/1000 respectively [27]. To reduce mortality rates among under-five children, increasing EBF and making sure the continuation of complementary foods for up to two years with milk feeding practice is the most feasible and scientific way to do so. A report in the Gambia said that Gambians have a low opinion of breastfeeding practices, and many of them follow traditional beliefs. As this poses a serious health problem for children, the government needs to improve the quality of health services, adapt them to the traditional and modern methods of breastfeeding, and mobilize people who play a significant role in society [28].

For Gambians, having a piece of current nationwide information about EBF rates and possible predictor factors is crucial in identifying public health priorities for key stakeholders in formulating, protecting, promoting, and adapting an effective integrated health program intervention. In conclusion, the study findings will inform decision-making practices that help governmental and non-governmental organizations, policymakers, researchers, health care providers, and their institutions improve EBF practices and subsequently reduce the high burden of under-five deaths in the country. This study targeted to evaluate EBF status and the potential individual and community-based factors in the Gambia using the Gambian 2019–20 DHS microdata.

## Methods and materials

### Study design and setting

This study was based on a secondary, large community-based survey, the Gambia Demographic Health Survey (GDHS), conducted from November 21, 2019, to March 30, 2020, in the Gambia. The 2019/20 Gambia Demographic and Health Survey (GDHS 2019/20), which was conducted by the Gambia Bureau of Statistics (GBoS) in coordination with the Ministry of Health and Social Welfare, took place from October 2019 to February 2020. The second DHS survey to be carried out in The Gambia in conjunction with the global Demographic and Health Survey Program is the Gambia Demographic and Health Survey (GDHS 2019–20). The Gambia is located on the West African coast. It is bordered on the north, south, and east by the Republic of Senegal, and on the west by the Atlantic Ocean. The country has a tropical climate characterized by two seasons: the rainy season (June–October) and the dry season (November–May) [29].

### Source population and sampling technique

The survey employed stratified, two-stage cluster sampling. In the first stage, enumeration areas (EAs) were selected with a probability proportional to their size within each sampling stratum. In the second stage, the households were systematically sampled. The source population was mothers who had infants. Only mothers with infants younger than 6 months were included. On the other hand, mothers whose children were not alive at the time of collection, those whose newborns did not live with them, and mothers whose children were over 6 months old were excluded from the study. The women’s sample weightings were used in the estimation to overcome disproportional allocations of samples during data collection. Accordingly, 897 weighted mother-infant pair samples were included in the study.

### Variables of the study

#### Dependent variable

The outcome of this study was the exclusive breastfeeding practice of mothers with infants under the age of six months. The variable was dichotomized into Yes = 1 or No = 0. “Yes = 1” was assigned to those mothers who stated that they had not given their child any food other than breast milk, while “No = 0” was assigned to those who stated that they had given their child any food other than breast milk.

#### Independent variables

Age of infants, sex of infants, birth order, breastfeeding initiation time, the child had a history of fever and diarrhea, birth weight, the child had ever drunk anything from a bottle with a nipple, age of mothers, marital status, educational level, occupation, ethnicity, type of residence, region, total children ever born, number of children under five, wealth status, media exposure, antenatal care and postnatal care visits, place of delivery, caesarian delivery, health provider checked mothers health after discharge; an assistant traditional birth attendant attended every birth in the last five years; a health provider counseled on breastfeeding and newborn dangers; a health provider observed breastfeeding.

### Data analysis

Data were extracted and analyzed using STATA version 14.0 statistical software. Descriptive studies like frequency count and proportion for categorical data were used to summarize the descriptive data. Bivariable logistic regression was used to select candidate variables for multiple logistic regression. In the bivariable logistic regression, a p-value of less than 0.2 was used as a cut point to select variables for the multiple logistic regression analysis entry. Multiple logistic regression was used to identify independent predictors of exclusive breastfeeding in the Gambia while controlling for confounders. 95% confidence interval (CI) and p-value 0.05 were used to determine the statistical significance. A logistic regression was fitted to assess multicollinearity among the independent variables using the variance inflation factor. Moreover, the Hosmer and Lemeshow test was used to assess the overall fitness of the final regression model.

## Results

### Sociodemographic characteristics of study participants

In the present study, a total of 897 weighted mothers and children under the age of six months were included. About 454 (50.63%) and 315 (35.13%) of children were male in sex and found in the age group of 0–1 month, respectively. Regarding early initiation of breastfeeding, only 297 (33.35%) of children took their mothers’ breasts soon after birth. About 384 (42.83%) children have average birth weight, and 416 (46.37%) have experienced both fever and diarrhea (Table 1).

**Table 1:** Characteristics of children under six months of age in Gambia, GDHS, 2019-20 (N = 897)

Variables		EBF prevalence		Total, n (%) Weighted	95% CI	P-value
		No, n (%)	Yes, n (%)			
Age of infant						
	0–1 month	79(25.21)	236(74.79)	315(35.13)		
	2–3 months	123(43.26)	161(56.74)	284(31.73)	0.45(0.32,0.63)	0.0001
	4–5 months	213(71.76)	84(28.24)	297(33.14)	0.13(0.08,0.18)	0.0001
Sex of infant						
	Male	218(48.05)	236(51.95)	454(50.63)		
	Female	197(44.64)	246(55.36)	443(49.37)	0.77,1.27)	0.940
Birth order						
	First	95(50.28)	94(49.72)	189(21.08)		
	2–3	153(48.77)	161(51.23)	314(35.01)	1.03(0.73,1.45)	0.886
	= >4	168(42.57)	226(57.43)	394(43.91)	1.29(0.93,1.79)	0.123
Breastfeeding initiation time						
	Immediately after birth	132(44.40)	165(55.60)	297(33.35)		
	Within hours	243(45.26)	294(54.74)	538(60.36)	0.99(0.76, 1.29)	0.954
	Within days	35(62.05)	21(37.95)	56(6.29)	0.62(0.35,1.10)	0.2103
The child had experienced a fever						
	No	338(81.32)	78(18.68)	416(46.37)		
	Yes	433(90.06)	48(9.94)	481(53.63)	0.52(0.37,0.72)	0.0001
The child had experienced diarrhea						
	No	327(78.65)	89(21.35)	416(46.37)		
	Yes	422(87.68)	59(12.32)	481(53.37)	0.58(0.41,0.81)	0.001
Birth weight						
	Very large	59(42.61)	80(57.39)	139(15.47)	1.02(0.71,1.46)	0.930
	Larger than average	99(47.29)	110(52.71)	209(23.36)	0.83(0.58,1.12)	0.192
	Average	168(43.63)	216(56.37)	384(42.83)		
	Smaller than average	60(56.44)	46(43.56)	106(11.86)	0.81(0.53,1.22)	0.305
	Very small	27(50.62)	27(49.38)	54(6.01)	0.68(0.39, 1.15)	0.152
	Don’t know	2.7(64.29)	1.5(35.71)	4.2(0.47)	1.32(0.12,14.61)	0.823
Child ever drunk anything from a bottle with a nipple						
	No	280(67.26)	136(32.74)	416(46.37)		
	Yes	466(96.83)	15(3.17)	487(53.63)	0.06(0.038,0.11)	0.0001

Nearly half of the mothers (444, or 49.53%) were estimated to be between 25 and 34 years old. Almost all mothers were in unions (844, or 94.12%), and nearly half of them had not gone through formal education (392, or 43.70%). Above half of them had no current jobs (516, or 57.59%), with the highest proportion of Mandinka/Jahanka in their ethnicity, at 296 (33.04%). According to region and type of resident, about 335 (37.32%) were from Brikama, and 558 (62.31%) of them were urban residents. Study participants have shown similar characteristics in terms of wealth status, with a low proportion of 148 (16.47%) and 199 (22.12) for the poorest and poorest, respectively (Table 2).

**Table 2:** Maternal socio-demographic and socioeconomic characteristics in Gambia, GDHS 2019-20 (n = 897)

Variables			EBF Prevalence		Total, n (%)	95% CI	P-value
			No, n (%)	Yes, n (%)			
Age of mother (years)							
	15–24		144(49.65)	146(50.35)	290(32.30)		
	25–34		197(44.30)	247(55.70)	444(49.53)	1.05(0.79,1.38)	0.752
	35–49		75(46.14)	88(53.86)	163(18.17)	1.08(0.75, 1.56)	0.664
Marital status							
	Never in union		28(53.87)	24(46.13)	53(5.88)		
	In union		387(45.90)	457(54.10)	844(94.12)	0.71(0.41,1.23)	0.23
Maternal education							
	No formal education		187(47.74)	205(52.26)	392(43.70)		
	Primary		71(46.63)	81(53.37)	153(17.03)	0.92(0.66,1.29)	0.631
	Secondary		142(45.48)	171(54.52)	313(34.96)	1.11(0.82,1.48)	0.509
	Higher		15(38.58)	24(61.42)	39(4.31)	1.10(0.52, 2.34)	0.801
Maternal occupation							
	Currently not working		169(44.50)	211(55.50)	380(42.41)		
	Currently working		246(47.74)	270(52.26)	516(57.59)	0.98(0.75,1.27)	0.870
Ethnicity							
	mandinka/jahanka		122(41.37)	174(58.63)	296(33.04)		
	Wollof		69(62.90)	41(37.10)	110(12.32)	0.41(0.27, 0.62)	0.001
	jola/karoninka		16(31.45)	36(68.55)	52(5.79)	1.14(0.59,2.62)	0.567
	fula/tukulur/lorobo		89(44.52)	111(55.48)	200(22.22)	0.82(0.58, 1.16)	0.265
	Serere		17(67.64)	8(32.36)	25(2.79)	0.24(0.09,0.66)	0.005
	Sarahule		38(45.05)	46(54.95)	84(9.35)	0.94(0.42,0.98)	0.042
	creole/aku marabout		2(79.85)	0.5(20.15)	2.5(0.28)	0.53(0.07, 3.80)	0.525
	Manjago		1.2(39.68)	2.0(60.32)	3.2(0.36)	0.53(0.07,3.79)	0.523
	Bambara		10(73.48)	4(26.52)	14(1.51)	0.33(0.11,1.03)	0.056
	Other		1(27.41)	4(72.59)	5(0.61)	1.58(0.16,15.36)	0.695
	non-Gambian		49(46.81)	56(53.19)	105(11.74)	0.64(0.41, 1.01)	0.051
Place of residence							
	Urban		296(53.03)	262(46.97)	558(62.31)		
	Rural		119(35.36)	219(64.64)	338(37.69)	1.84(1.43, 2.38)	0.0001
Region							
	Banjul		4(51.05)	3(48.95)	7(0.83)		
	Kanifing		834(55.76)	66(44.24)	150(16.74)	1.02(0.48,2.12)	0.966
	Brikama		172(51.55)	162(48.45)	335(37.32)	1.21(0.60, 2.41)	0.597
	Mansakonko		14(38.28)	23(61.72)	37(4.08)	1.93(0.91,4.08)	0.086
	Kerewan		48(39.63)	74(60.37)	122(13.65)	1.79(0.89,3.58)	0.102
	Kuntaur		23(42.93)	31(57.07)	54(6.03)	1.68(0.83,3.40)	0.146
	Janjanbureh		21(31.71)	46(68.29)	67(7.44)	2.39(1.18, 4.85)	0.016
	Basse		49(39.18)	76(60.82)	125(13.92)	1.70(0.86,3.32)	0.124
Total children ever born							
	< 5		302(47.80)	329(52.20)	631(70.38)		
	5–6		74(45.74)	87(54.26)	161(17.95)	1.17(0.84, 1.63)	0.363
	7–8		24(31.65)	51(68.35)	75(8.38)	1.58(1.01,2.48)	0.044
	= 9+		17(56.50)	13(43.50)	30(3.28)	0.77(0.41,1.45)	0.417
Number of under-five children							
	0–2		176(50.56)	172(49.44)	349(38.89)		
	3–5		164(43.16)	216(56.84)	380(42.34)	1.24(0.94, 1.65)	0.134
	5+		75(44.89)	93(55.11)	168(18.76)	1.29(0.92,1.84)	0.144
Household wealth index							
	Poorest		66(33.27)	132(66.73)	199(22.13)		
	Poorer		103(51.97)	96(48.03)	199(22.12)	0.62(0.44, 0.87)	0.006
	Middle		72(40.34)	106(59.66)	178(19.86)	0.82(0.57,1.18)	0.295
	Richer		81(54.52)	67(45.48)	148(16.47)	0.48(0.32,0.73)	0.001
	Richest		94(54.16)	79(45.84)	173(19.32)	0.54(0.35,0.83)	0.005
Media exposure							
	Listening to radio	No	260(46.53)	299(53.47)	559(62.37)		
		Yes	156(46.09)	181(53.91)	337(37.63)	0.94(0.73, 1.21)	0.635
	Watching to TV	No	179(43.15)	271(56.25)	450(49.83)		
		Yes	236(56.85)	210(43.75)	447(50.17)	0.67(0.52, 0.86)	0.002
	Reading to newspaper	No	413(46.78)	469(53.22)	882(98.41)		
		Yes	3(20.61)	12(79.39)	15(1.59)	2.68(0.74, 9.68)	0.131

Regarding maternal obstetric and related healthcare characteristics, 682 (76.08%), 795 (88.67%), and 860 (95.96%) mothers had more than four antenatal care follow-ups during their pregnancy, had given birth to their children at health facilities, and had no history of caesarian section delivery, respectively. All mothers had given birth in the last five years, and nearly half of them (53.6%) had postnatal care visits. About half of 441 (49.27%) and 473 (52.76%) mothers had and had not received breastfeeding counseling and information about dangerous signs of newborns from the health care providers, respectively (Table 3).

**Table 3:** Maternal obstetric and healthcare-related characteristics in the Gambia, GDHS 2019-20 (n = 897)

Variables		EBF prevalence		Total, n (%)	95% CI	P-value
		No, n (%)	Yes, n (%)			
ANC visit						
	No visit	5(55.97)	4(44.03)	9(1.04)		
	1 visit	13(59.61)	9(40.39)	22(2.45)	0.57(0.18,2.17)	0.406
	2–3 visits	104(56.65)	79(43.35)	183(20.44)	0.83(0.29, 2.31)	0.718
	4 + visits	294(43.05)	388(56.95)	682(76.08)	1.17(0.43,3.17)	0.575
Place of delivery						
	Home	34(33.37)	68(66.63)	102(11.63)		
	Health facility	382(48.03)	413(51.97)	795(88.67)	0.56(0.38,0.81)	0.002
Caesarian delivery						
	No	394(45.79)	466(54.21)	860(95.96)		
	Yes	22(59.94)	14(40.06)	36(4.04)	0.67(0.33, 1.37)	0.272
Respondent’s health check after discharge/delivery at home						
	No	300(50.37)	296(49.63)	596(66.53)		
	Yes	115(38.40)	185(61.60)	300(33.47)	1.21(0.94,1.56)	0.140
Assistance traditional birth attendant						
	No	401(47.49)	443(52.51)	844(94.10)		
	Yes	15(28.49)	38(71.51)	53(5.90)	1.91(1.16,3.11)	0.01
PNC visit						
	No	181(43.57)	235(56.43)	387(46.37)		
	Yes	206(42.82)	275(57.18)	507(53.63)	0.99(0.76,1.28)	0.598
Births in the last five years						
	1	162(50.05)	162(49.95)	324(36.14)		
	2–3	253(44.46)	316(55.54)	568(63.40)	1.32(1.02, 1.72)	0.036
	4–5	0.8(18.51)	3.3(81.49)	4.1(0.46)	2.17(0.42,11.37)	0.356
Health provider counsel on breastfeeding						
	No	215(47.34)	240(52.66)	455(50.73)		
	Yes	200(45.36)	241(45.36)	441(49.27)	1.21(0.94, 1.55)	0.149
Health providers observe breastfeeding						
	No	226(47.18)	253(47.18)	480(53.49)		
	Yes	189(45.42)	228(54.58)	417(46.51)	1.12(0.87,1.44)	0.364
Health provider counsel on newborn dangers						
	No	222(46.95)	251(63.05)	473(52.76)		
	Yes	194(45.71)	230(54.29)	424(47.24)	1.11(0.86,1.43)	0.419

### Factors associated with exclusive breastfeeding

In the logistic multiple regression analysis, the age of the child, type of residence, exposure to the reading newspaper, health providers counseling on breastfeeding, and experienced fever was among the significant factors associated with participant’s practice of exclusive breastfeeding under six months of age children in the Gambia.

Children within the age range of 2–3 months (Adjusted Odds Ratio (AOR = 0.41, 95 CI% (0.28, 0.59)) and 4–5 months (Adjusted Odds Ratio (AOR = 0.11, 95% CI: (0.07, 0.16)) were less likely to be feed breastfeeding exclusively compared to participants with the age range of 0–01 month of children. Mothers living in rural areas of the Gambia have (Adjusted Odds Ratio (AOR = 2.14, 95% CI: 1.33,3.41) times more experience in being fed their children exclusively than those participants who have lived in the urban areas of the country. On the other hand, those mothers who have the exposure of reading to the newspaper have (Adjusted Odds Ratio (AOR = 5.62, 95% CI: 1.32,24.09) times more tendency to continue exclusively breastfeeding than mothers who have not read the newspaper. Participants who have received breastfeeding counseling from health providers have (Adjusted Odds Ratio (AOR = 1.36, 95% CI: 1.01,1.82) times being exclusive breastfeeder mothers compared to mothers who did not receive any counseling from health providers on breastfeeding. The last but not least factor was fever. Mothers whose children had experienced fever have less likely to be not exclusive feeder parents than those whose child did not experience a fever by (Adjusted Odds Ratio (AOR = 0.56, 95% CI: 0.37,0.84) (Table 4).

**Table 4:** Factors associated with Exclusive breastfeeding among under six months aged children in the Gambia, GDHS 2019-20(n = 897)

Variables		Exclusive breastfeeding		COR (95%)		AOR (95%)	p-value
		No, n (%)	Yes, n (%)				
Age of infant							
	0–1 month	79(25.21)	236(74.79)	1		1	
	2–3 months	123(43.26)	161(56.74)	0.45(0.32,0.63)		0.41(0.28,0.59)	0.0001
	4–5 months	213(71.76)	84(28.24)	0.13(0.08,0.18)		0.11(0 0.07,0.16)	0.0001
Birth order							
	First	95(50.28)	94(49.72)	1		1	
	2–3	153(48.77)	161(51.23)	1.03(0.73,1.45)		0.74(0.43,1.27)	0.272
	= 4+	168(42.57)	226(57.43)	1.29(0.93,1.79)		0.91(0.48,1.72)	0.777
Marital status							
	Never in union	28(53.87)	24(46.13)	1		1	
	In union	387(45.90)	457(54.10)	1.41(0.82,2.44)		1.29(0.68,2.47)	0.392
Place of delivery							
	Home	34(33.37)	68(66.63)	1		1	
	Health facility	382(48.03)	413(51.97)	0.56(0.38,0.81)		0.64(0.36,1.15)	0.138
Place of residence							
	Urban	296(53.03)	262(46.97)	1		1	
	Rural	119(35.36)	219(64.64)	1.84(1.43,2.37)		2.14(1.33,3.41)	0.001
Region							
	Banjul	4(51.05)	3(48.95)	1		1	
	Kanifing	834(55.76)	66(44.24)	1.02(0.49,2.13)		1.34(0 0.58,3.08)	0.498
	Brikama	172(51.55)	162(48.45)	1.21(0.60,2.41)		1.44(0.65,3.21)	0.369
	Mansakonko	14(38.28)	23(61.72)	1.93(0.91,4.08)		1.65(0 0.65,4.19)	0.294
	Kerewan	48(39.63)	74(60.37)	1.79(0.89,3.58)		1.62(0.68,3.87)	0.278
	Kuntaur	23(42.93)	31(57.07)	1.68(0.83,3.40)		1.12(0.44,2.83)	0.808
	Janjanbureh	21(31.71)	46(68.29)	2.39(1.18,4.85)		1.94(0.81,4.71)	0.141
	Basse	49(39.18)	76(60.82)	1.69(0.86,3.33)		1.85(0.79,4.32)	0.156
Total children ever born							
	< 5	302(47.80)	329(52.20)	1		1	
	5–6	74(45.74)	87(54.26)	1.17(0.84,1.63)		0.97(0.58,1.64)	0.921
	7–8	24(31.65)	51(68.35)	1.58(1.01,2.48)		1.22(0.65,2.29)	0.532
	= 9+	17(56.50)	13(43.50)	0.77(0.41,1.45)		0.48(0.21,1.11)	0.087
Wealth status							
	Poorest	66(33.27)	132(66.73)	1		1	
	Poorer	103(51.97)	96(48.03)	0.62(0.44,0.87)		0.75(0.48,1.15)	0.186
	Meddle	72(40.34)	106(59.66)	0.82(0.57,1.18)		1.31(0.75,2.28)	0.339
	Richer	81(54.52)	67(45.48)	0.48(0.32,0.73)		0.90(0.47,1.74)	0.757
	Richest	94(54.16)	79(45.84)	0.54(0.35,0.83)		1.03(0.49,2.15)	0.939
Number of under-five children							
	0–2	176(50.56)	172(49.44)	1		1	
	3–5	164(43.16)	216(56.84)	1.33(1.02,1.72)		1.25(0.79,1.95)	0.588
	5+	75(44.89)	93(55.11)	2.17(0.42,11.37)		1.03(0.17,6.07)	0.833
Watching television							
	No	179(43.15)	271(56.25)	1		1	
	Yes	236(56.85)	210(43.75)	0.67(0.52,0.86)		0.78(0.55,1.10)	0.157
Reading to newspaper							
	No	413(46.78)	469(53.22)	1		1	
	Yes	3(20.61)	12(79.39)	2.68(0.74,9.68)		5.62(1.32,24.09)	0.020
Assistance TBAs							
	No	401(47.49)	443(52.51)	1		1	0.779
	Yes	15(28.49)	38(71.51)	1.90(1.16,3.11)		1.11(0.52,2.37)	
Births in the last five years							
	1	162(50.05)	162(49.95)	1		1	
	2–3	253(44.46)	316(55.54)	1.33(1.02,1.72)		1.25(0.79,1.95)	0.330
	4–5	0.8(18.51)	3.3(81.49)	2.18(0.42,11.37)		1.03(0.17,6.06)	0.972
The health provider counseled on breastfeeding							
	No	215(47.34)	240(52.66)	1		1	
	Yes	200(45.36)	241(45.36)	1.20(0.94,1.55)		1.36(1.01,1.82)	0.044
The child had experienced a fever							
	No	338(81.32)	78(18.68)	1		1	
	Yes	433(90.06)	48(9.94)	0.52(0.37,0.72)		0.56(0.37,0.84)	0.005
The child had experienced diarrhea							
No		327(78.65)	89(21.35)	1		1	
Yes		422(87.68)	59(12.32)	0.58(0.41,0.81)		1.01(0.67,1.52)	0.954

*A p-value of 0.0001 indicates p < 0.000

### Prevalence of exclusive breastfeeding in the Gambia

The overall prevalence of EBF among under-six-month-old children in the Gambia was found to be low; only 481 (53.63%, 95% CI: 50.35, 56.88) of them had practiced EBF.

## Discussion

This study aimed to assess the status of exclusive breastfeeding practice among mothers who have children under the age of six months, and it declared that the overall practice of exclusive breastfeeding among under-six-month-old children in the Gambia remains low among mothers who have experienced EBF. The proportion of participants in this study was higher with studies conducted in Ghana 43.7% [30], Cameroon 45.2% [31], Kenya 33% [32], Malaysia 41.7% [33], Nigeria [34], and Gambia [25]. However it is lower than studies done in Ethiopia 57.3% [35], and India [36], and it is almost in agreement with a study done in Bangladesh 56% [37] Pakistan 53.6% [38], and a systematic review of east African countries 55.9% [11]. The empirical findings of this study may be less than those of the previous one due to national profiles of EBF experience, government policy, and closed follow-up on EBF, children’s health, and participants’ culture, attitude, knowledge, and education backgrounds concerning EBF. On the other hand, this study discovered a substantially greater prevalence of EBF, which could be due to several factors such as limiting confounding factors both at the individual and community levels, methods of recruiting participants, time variation, and other methodological difficulties. This could have influenced their prevalence findings.

The study at hand discovered that the age of a child was one predictor factor of exclusive breastfeeding after controlling other variables. Children whose ages are between 2 and 3 months and 4–5 months have a lower likelihood of being exposed during a stay at EBF than children aged 0–1 month. This figure is consistent with studies done in Ghana [30], Guinea [39], Ethiopia [40] and Canada [41]. This results in the fact that as children get closer to six months of age, they are less likely to take EBF alone. This is a matter of precaution and scientific acceptance. By the time they are six months old, it is common and expected for mothers to feed their babies on regular basis in addition to mothers’ milk.

The study also revealed that mothers from rural areas of the Gambia have more likelihood of feeding their children exclusively than participants living in urban areas. The figure of this study had in agreement with plenty of other studies done in various settings in the 13 economic communities of west African states (ECOWAS) countries [42]. The finding could be explained by mothers who are from rural areas, who might have no chance of accessing infant formula feeding due to the cost, and availability of it. Because those countryside participants might have a less powerful influence against their husbands/community on various issues both economic and political. On the other hand, those rural area residents might have faced knowledge and practice gaps to prepare infant formula or other complementary foods to feed their children. Last but not least there is a usual occasion that, when mothers provide infant formula or any other complementary foods to their children due to children’s digestive systems, children might have a probability of diarrhea and other unexpected health problems [31]. At this stage, those mothers might be perceived that the illness of the child is due to the complementary foods that they fed, and they will not provide any complementary foods then. On the contrary, mothers living in urban areas, on the other hand, are more likely to work outside the home, either in government or in private, so they may not have enough time to breastfeed their children, and the duration of maternity leave is a very short time, especially, in those ECOWAS countries, this remains a public challenge [17, 43]. For instance in Nigeria maternity leave duration is 16 weeks only [43], Ghana 12 weeks only [44]. Furthermore, these mothers are better off financially, so they have more money with access and availability to buy infant formula feeding and other complementary foods for their children and more self-determination [31, 40].

The other exciting variable was reading the newspaper. The study at hand depicted those mothers who read newspapers have more chance of being exclusive breast feeders to their children. The result could be justified by mothers who have read newspapers for at least a week and might receive much information about the advantage of EBF for both the child and the mother. This is because, first of all, these mothers are educated and probably have a better income. Alternatively, the benefits of breastfeeding may be exclusively covered in the newspaper. A time series study in the USA from 19,972 to 2000 showed that media exposure such as reading magazines has a great potential of changing the mind of mothers regarding EBF [45, 46], in Guinea [47], and in Ghana [48].

Participants who have received breastfeeding counseling from health providers have more experienced EBF than their counterparts. This study has the same findings as studies done in Ghan a[30], Gambia [25], and a systematic review report [49]. This scenario may be because health professionals may have more profound knowledge and positive attitude than many mothers think about the benefits and harms of breastfeeding. As a result, counseled mothers are determined to continue EBF giving their children the advice of health care providers to keep their children healthy, active and competitive in the schools and communities as they received from the professionals.

Mothers whose children had experienced fever have less chance of continuing EBF than their counterparts. This finding has shown that mothers have both knowledge and practice as well as attitude gap barriers by the time their children are under the weather by fever. These mothers are in a knowledge deficit and can give their children everything they accessed at home when their children become febrile. On the other side, those children might a greater chance of exposure to fever, while they are not put on EBF. Because studies showed that a lack of EBF increased the odds of dehydration, fever and diarrhea, and other infections [37, 38, 48]. However, if they were taken to a health institution, health providers would not only treat the fever but also give them scientific advice on how to maintain their exclusive breastfeeding instead of eating extra meals when they have such problems. Mothers might have a knowledge deficit that their milk has an antibody to the child.

This study’s use of a sizable sample size is one of its strengths. The likelihood of departures from the actual population declining as sample size rises also makes it more likely that it will be trustworthy for use by subsequent researchers. Additionally, the right statistical model that can consider the nature of the data was used. Although this study considered several significant response variables, we were unable to analyze study participants’ HIV status since there is no record of their HIV status for exclusive breastfeeding in The Gambia. Considering HIV status may therefore be necessary given the context. Certain variables or confounders, such as the HIV status of the study participants, are missed in the analysis because it is reliant on the information in the data set. Last but not least, future researchers may find it valuable to do additional primary research by using a variety of methods and designs to determine HIV status and other relevant characteristics of exclusive breastfeeding. The cross-sectional design of the study precludes concluding the causes of the relationships that were found, hence the present study should be regarded with caution. Moreover, because all of the data were self-reported, social desirability bias could have been introduced.

## Conclusion

This study assessed the status of EBF in the Gambia among children under the age of six months, and it found the figure substantially unsuccessful and remaining low in the Gambia. The age of the child, being a rural resident, having exposure from reading the newspaper, mothers who have received health care provider counseling on breastfeeding, and a child being exposed to fever were among the factors that were statistically significant after adjusting and controlling for possible confounders in the multivariable regression analysis. It is crucial to educate and create awareness on the importance of exclusive breastfeeding and to advise mothers to take their children to a health facility in case of illness and not to stop exclusive breastfeeding at the time of their illness. The government of Gambia may implement policies both in urban and rural areas of the country accordingly to avert the issue of unsuccessful exclusive breastfeeding practices.

## Data Availability

Data for this study were obtained from the publicly available DHS Program on Gambian 2019/20 DHS data files. The datasets generated and/or analyzed during the current study are available in the DHS program repository at www.dhsprogram.com.

## Declarations

We hereby declare that this EBF study represents our work which has been done after registration and reception for the accessing of data at www.dhsprogram.com and confirms that all methods were carried out following relevant guidelines and regulations. We have read the journal’s current research ethics guidelines, and accept responsibility for the conduct of the procedures under the journals. We have attempted to identify all the risks related to this research that may arise in conducting this research, obtained the relevant ethical and/or safety approval (where applicable), and acknowledged my obligations and the rights of the participants by the demography and health survey program authorities. The research is conducted by the declaration of Helsinki.
